# FDG and FLT-PET for Early measurement of response to 37.5 mg daily sunitinib therapy in metastatic renal cell carcinoma

**DOI:** 10.1186/s40644-015-0049-x

**Published:** 2015-09-03

**Authors:** Kevin P. Horn, Jeffrey T. Yap, Neeraj Agarwal, Kathryn A. Morton, Dan J. Kadrmas, Britney Beardmore, Regan I. Butterfield, Kenneth Boucher, John M. Hoffman

**Affiliations:** Center for Quantitative Cancer Imaging, Huntsman Cancer Institute, University of Utah, 1950 Circle of Hope Dr, Suite 6810, Salt Lake City, UT 84112-5560 USA; Department of Radiology, University of Utah, 30 North 1900 East #1A071, Salt Lake City, UT 84132-2140 USA; Department of Medicine, Huntsman Cancer Institute, University of Utah, 2000 Circle of Hope Dr, Salt Lake City, UT 84112-5550 USA; Athinoula A. Martinos Center for Biomedical Imaging, Massachusetts General Hospital, 149 Thirteenth Street, Suite 2301, Charlestown, MA 02129 USA; Department of Internal Medicine, Huntsman Cancer Institute, University of Utah, 2000 Circle of Hope Dr, Salt Lake City, UT 84112-5550 USA

**Keywords:** ^18^ F-fluorodeoxyglucose (FDG), ^18^ F-fluorothymidine (FLT), Positron emission tomography (PET), Renal cell carcinoma, Response assessment, Sunitinib, Tumor metabolism, Tumor proliferation

## Abstract

**Background:**

Metastatic renal cell carcinoma has a poor prognosis and an intrinsic resistance to standard treatment. Sunitinib is an oral receptor tyrosine kinase inhibitor that has been used as a first-line targeted therapy in metastatic renal cell carcinoma. While computed tomography (CT) is currently the gold standard for response assessment in oncological trials, numerous studies have shown that positron emission tomography (PET) imaging can provide information predictive of tumor response to treatment earlier than the typical interval for standard of care follow-up CT imaging. In this exploratory study we sought to characterize early tumor response in patients with metastatic renal cell carcinoma treated with continuous daily 37.5 mg sunitinib therapy.

**Methods:**

Twenty patients underwent dynamic acquisition positron emission tomography (PET) imaging using ^18^ F-fluorodeoxyglucose (FDG) and ^18^ F-fluorothymidine (FLT) at baseline and early in treatment (after 1, 2, 3 or 4 weeks) with 37.5 mg continuous daily dosing of sunitinib. Semi-quantitative analyses were performed to characterize the tumor metabolic (FDG) and proliferative (FLT) responses to treatment.

**Results:**

Proliferative responses were observed in 9/19 patients and occurred in 2 patients at one week (the earliest interval evaluated) after the initiation of therapy. A metabolic response was observed in 5/19 patients, however this was not observed until after two weeks of therapy were completed. Metabolic progression was observed in 2/19 patients and proliferative progression was observed in 1/19 patients. Baseline FDG-PET tumor maximum standardized uptake values correlated inversely with overall survival (*p* = 0.0036). Conversely, baseline ^18^ F-fluorothymidine PET imaging did not have prognostic value (*p* = 0.56) but showed a greater early response rate at 1–2 weeks after initiating therapy.

**Conclusions:**

While preliminary in nature, these results show an immediate and sustained proliferative response followed by a delayed metabolic response beginning after two weeks in metastatic renal cell carcinoma treated with a continuous daily dose of 37.5 mg sunitinib. The results provide evidence of tumor response to lower-dose sunitinib while also supporting the inclusion of PET imaging as a tool for early assessment in oncological clinical trials.

**Trial registration:**

ID: NCT00694096

## Background

Sunitinib, a tyrosine kinase inhibitor that inhibits VEGF receptor (types 1–3) and PDGF receptor (α and β), as well as other tyrosine protein kinases;[1–4] is an approved treatment of metastatic renal cell carcinoma (RCC). In an early phase I trial including 4 patients with metastatic RCC, an objective response was observed in 3 out of those patients [5]. Phase II studies of sunitinib in 63 and 106 metastatic RCC patients revealed a partial response rate of 36-40 % and a progression-free survival of 8.3-8.7 months [6, 7]. A randomized phase III trial has shown sunitinib to be superior to interferon-α in treatment naïve patients with an objective response rate of 47 % (compared to 12 %) and a progression free survival of 11 months (compared to 5 months) [8]. In the intermediate-risk group, median overall survival was 20.7 months with the treatment of sunitinib compared to 15.4 months with interferon-α while the poor-risk group had a median survival of 5.3 months with sunitinib compared to 4.0 months with interferon-α.

X-ray computed tomography (CT) is currently the gold standard for response assessment in oncological clinical trials and clinical practice [9, 10]. However, positron emission tomography (PET) imaging with ^18^ F-fluorodeoxyglucose (FDG) is also well-validated in the diagnosis, staging, and response assessment of patients with cancer as the majority of malignant tissues have increased FDG uptake associated with an increased rate of glycolysis as well as increased glucose transport [11, 12]. FDG-PET has been valuable in the characterization and primary staging of solid renal masses visualized by CT or MRI [13] and in the detection of distant metastasis in renal cancer [14]. For the evaluation of metastases, investigations have found a range of sensitivities from 60 % to 87 % and specificities close to 100 %, [15–17] with a positive scan being predictive of the presence of disease in suspected metastases [18]. Finally, FDG-PET has already shown efficacy for early response assessment in therapeutic trials, including sunitinib therapy in gastrointestinal stromal tumors [19].

Specifically in RCC, FDG-PET has shown utility in predicting response to sunitinib and other tyrosine kinase inhibitors in RCC when baseline studies are compared to those after 4–16 weeks of treatment [20–23]. Farnebo, et al. evaluated early response of metastatic RCC to treatment with tyrosine kinase inhibitors using FDG-PET after 2 or 4 weeks of therapy and included 19 patients who received sunitinib at 50 mg/day [24]. This study did show that FDG-PET could successfully assess response at 2 weeks, however, data from all tyrosine kinase inhibitors were combined and analysis of patients only receiving sunitinib was not reported.

^18^ F-fluorothymidine (FLT) is a structural analog of the DNA constituent thymidine, which is not incorporated into DNA, but instead is trapped intracellularly after phosphorylation by thymidine kinase [25]. As such, FLT has significant potential as an experimental radiolabeled imaging agent to evaluate tumor cellular proliferation with PET, [26, 27] To date, FLT has been utilized in numerous published oncology trials to evaluate response to treatment [28] and is known to have uptake in RCC [29]. A study by Liu, et al. imaged 16 patients (seven with metastatic RCC) receiving sunitinib therapy at baseline, the end of a treatment cycle, and the end of drug withdrawal cycle [30]. Sunitinib was administered in either a 4 weeks on/2 weeks off or a 2 weeks on/1 week off schedule. This study showed a reduction of proliferation during treatment with marked increase in cellular proliferation during the withdrawal phase.

Ideally, early phase clinical trials with novel targeted chemotherapies should include investigation of the optimal imaging strategy and timing for early assessment of response to the novel therapeutic agents. The two previous studies to demonstrate the ability of FDG-PET [24] and FLT-PET[30] to predict early response of metastatic RCC to sunitinib administered with the standard dosing regimen of 50 mg/day, 3 weeks on/1 week off. Here we evaluate the utility of FDG-PET and FLT-PET for early assessment of metabolic and proliferative response to treatment with sunitinib in metastatic RCC using 37.5 mg/day continuous dosing.

## Methods

### Study design

This exploratory clinical trial (clinicaltrials.gov ID: NCT00694096) was performed exclusively at the Huntsman Cancer Institute, University of Utah with institutional review board approval. All patients signed informed consent prior to enrollment. Patients were randomized to a 1, 2, 3, or 4 week treatment cohort at the time of consent. Upon enrollment, the Response Evaluation Criteria in Solid Tumors (RECIST 1.0) criteria [10] were used to select appropriately sized target lesions from clinical CT and/or magnetic resonance imaging (MRI) studies for further evaluation with PET. In addition, the PET target lesions were also required to be above background activity. Immediately after the completion of baseline PET imaging, treatment was initiated with continuous oral sunitinib at a dose of 37.5 mg/day. Early evaluation of disease response was performed by FDG-PET and FLT-PET after approximately 1, 2, 3, or 4 weeks of continuous daily treatment. Note that each patient only participated in a single post-treatment scan so there were no intra-patient comparisons at different time points.

### Eligibility criteria

Inclusion criteria included: aged 18 or older, previous histological diagnosis of RCC, documented evidence of metastatic disease on contrast-enhanced CT and/or MRI within 30 days with at least one measurable lesion, [10] and suitable candidacy for standard sunitinib therapy for metastatic RCC as determined by the treating oncologist. Exclusion criteria included: pregnancy or lactation (a negative pregnancy test was required for premenopausal females); renal insufficiency; known allergic or hypersensitivity reactions to previously administered radiopharmaceuticals; HIV positivity (as a precaution due to potential toxicity observed with 3'-deoxy-3'-fluorothymidine (alovudine) in therapeutic trials of HIV positive individuals [31]), and the requirement of monitored anesthesia sedation for PET scanning.

### Radiopharmaceuticals

FDG was manufactured locally in the PET Cyclotron Radiochemistry Laboratory at the Huntsman Cancer Institute under the practice of medicine and pharmacy. FLT was synthesized by methods previously reported [32, 33] and the FLT studies were conducted under an FDA investigational new drug application held by JMH.

### PET Imaging

All PET data were acquired using an Advance PET scanner (GE Healthcare, Milwaukee, WI) in 2D mode in order to minimize scatter and dead time. The patients were positioned such that the target lesion(s) were within a single 14.5 cm axial field of view. PET transmission scans were acquired for 5–10 min using a rotating ^68^Germanium rod source in order to perform attenuation correction. Following the transmission scan, 277.5 MBq (7.5 mCi) of FDG or 185 MBq (5 mCi) of FLT was infused intravenously via a peripheral vein over a period of one minute followed by a saline flush in the arm opposite that from which blood samples were subsequently drawn. At the time of radiotracer injection, a 70 min dynamic PET emission scan was performed for a single bed position to facilitate future exploratory kinetic analysis. A 20 min summed image was generated from the dynamic images and used for the SUV analysis in this study. Acquisitions were performed in 2D mode with the exception of the baseline FLT-PET scan for patient #9, which was inadvertently performed in 3D and was excluded from the analysis. FDG- and FLT-PET scans were performed on separate days, and typically on consecutive days. Patients were fasted for 6 h prior to FDG scans to reduce physiologic uptake in normal tissue. FLT-PET scans were scheduled on the first day of imaging and patients were also fasted to provide the opportunity for FDG-PET imaging in the event of failed FLT production, as well as to limit patient confusion in regard to preparation for the scanning session.

### FDG-PET and FLT-PET Semi-quantitative analysis

FDG-PET and FLT-PET images were reconstructed using ordered-subsets expectation-maximization (OSEM) with 2 iterations and 28 subsets, and no post-reconstruction filter. In one case (follow-up FDG-PET for patient# 16) reconstruction and raw data was lost due to computer failure. Static images were computed using summed data from the final dynamic time frames obtained at 50–70 min post-injection, representing a 60 min uptake period with a +/−10 min window, which is consistent with the NCI consensus recommendations for measurement of standardized uptake values (SUVs) in response assessment [12]. A region-of-interest (ROI) was drawn around each target lesion previously identified on clinical CT or MRI that was contained within the single PET bed position and confirmed to have FDG and FLT uptake above background. Due to the high liver uptake in FLT-PET studies, no liver lesions were selected. Each subject had a minimum of 1 target lesion on both FDG-PET and FLT-PET and up to a maximum of 6 lesions. Side-by-side image review and analysis was performed to assure that the regions were obtained from the same lesions on baseline and follow-up studies. Total body mass-corrected SUVs were calculated for each ROI by normalizing for injected activity and body weight as follows: SUV = tissue concentration (Bq/ml) X weight (g) / Dose (Bq). The maximum SUV (SUV_max_) for each ROI was determined. In individuals with multiple target lesions, SUV_max_ values from each ROI were summed to obtain a summed SUV_max_, analogous to the RECIST summed longest diameter [10]. This value was then divided by the number of target lesions to obtain the average SUV_max_ Metabolic response was assessed at the follow-up PET scans using EORTC response criteria based on the change in the follow-up average SUV_max_ relative to baseline as follows: Progressive Disease (PD) ≥ 25 % increase, Partial Response (PR) ≥ 25 % decrease, and Stable Disease (SD) < 25 % change [34]. Similarly, proliferative response were defined using the same +/−25 % thresholds based on the relative changes in the FLT average SUV_max_.

### Statistical analysis

The goal of the statistical analysis was to evaluate baseline FDG-PET and FLT-PET variables to overall survival and compare differences in early versus late response rates across the different cohorts with both PET radiopharmaceuticals. Overall survival was measured from the beginning of treatment. The per-patient average of the baseline FDG and FLT SUV_max_ was calculated by dividing the summed SUV_max_ of all target lesions for a given subject by the number of lesions. The total data set has 20 subjects with 4, 7, 5 and 4 subjects in the 1, 2, 3 and 4-week treatment cohorts respectively. All cohorts were analyzed together with clustering by cohort as the sample sizes were too small to analyze the cohorts separately. Cox proportional hazards models with robust standard errors accounting for small sample sizes [35] were used to analyze overall survival. The two predictor variables analyzed in this study consisted of baseline average SUV_max_ for both FDG-PET and FLT-PET. These predictor variables were treated as continuous predictors. The Benjamini and Hochberg method was used to adjust the p-values for multiple comparisons [36]. Kaplan-Meier methods were used to plot overall survival with the predictors dichotomized at the median. Logistic regression and the associated likelihood ratio test were used to test for a trend in response with group. Group was considered as a continuous variable with values 1, 2, 3, and 4 representing the weeks of treatment for each cohort. The statistical analysis was performed using R 3.0.2 statistical computing software (R development Core Team, 2013) [37].

## Results

### Patient population

From November 2007 to March 2012, 25 patients were enrolled in this study at the University of Utah Huntsman Cancer Institute. Twenty patients completed PET imaging at both time points and were considered evaluable for this study (completed both baseline and follow-up PET imaging assessments). Of the 5 patients not considered evaluable, one was unable to complete a 70 min imaging session due to pain and 3 did not return for post-treatment PET imaging due to transfer of care or being lost to follow-up. The one remaining patient did complete both PET imaging sessions, however, was found to have pathologically proven sarcoidosis at the location of PET imaging after undergoing surgical resection one week after completing the follow-up PET imaging assessment. The 20 patients included in this study were composed of 14 (70 %) males aged 50—76 (mean 63.6) years and 6 females aged 67—86 (mean 75) years (Table 1). One to six (mean 2.3) target lesions were identified per patient on clinical CT or MRI using RECIST 1.0 size criteria [10] and evaluated with subsequent PET imaging. Eighteen patients were naïve to prior systemic therapy prior to this study with the remaining two patients having failed IL-2 therapy. Fifteen patients had undergone nephrectomy prior to enrolling into this trial. FLT-PET and FDG-PET imaging studies were almost exclusively performed on consecutive days, however in two instances baseline FDG and FLT scans were performed 4 and 7 days apart due to patient scheduling conflicts and technical difficulties with radiotracer production, respectively. Four individuals had documentation of missed doses of sunitinib during the study period; the total number of missed doses for those individuals were: 1, 2, 2.5, and 5. The number of days on treatment and overall survival are summarized in Table 1. Due to drug-related adverse events and other clinical factors, many patients did not continue therapy for the total duration of their survival after the completion of imaging. As a result, survival analysis could not be performed using the post-treatment imaging results as a predictive biomarker.

**Table 1:** Study patient population and clinical outcome

Subject #	Age	Sex	Tumor Subtype	Therapy duration (days)	Survival (days)
14	67	F	Clear Cell	1518	1687
24	62	M	Clear Cell	565	1040
20	63	M	Clear Cell	1390	1390
5	60	M	Clear Cell	56	480
2	50	M	Mixed	9	2399
21	59	M	Clear Cell	1145	1390
22	65	M	Papillary	205	347
19	57	M	Clear Cell	84	262
23	67	F	Papillary	1064	1207
25	71	M	Clear Cell	27	27
18	76	F	Clear Cell	10	216
9	72	F	Mixed	33	98
15	86	F	Clear Cell	25	142
16	59	M	Clear Cell	29	183
11	70	M	Chromophobe	735	1756
17	68	M	Clear Cell	118	463
6	61	M	Clear Cell	400	1519
8	76	M	Clear Cell	133	791
10	69	M	Clear Cell	141	803
4	82	F	Unknown	30	544

### Early assessment of metabolic response with semi-quantitative FDG-PET

**Fig. 1:**
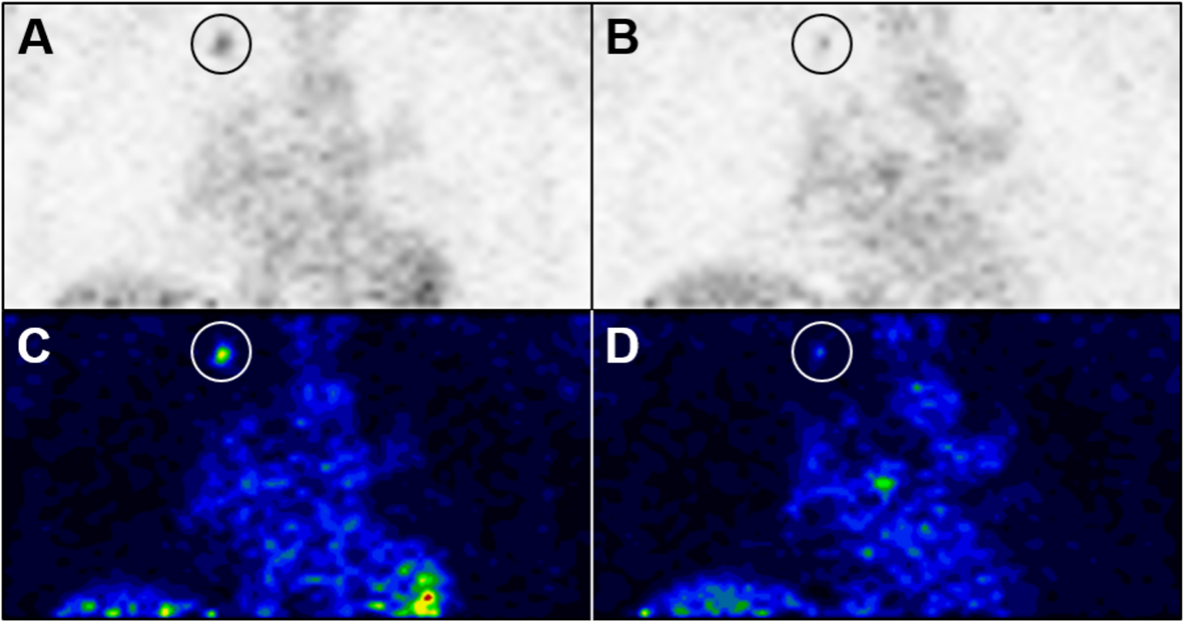
FDG-PET images illustrating a metabolic response to 37.5 mg daily sunitinib treatment. Single slice coronal inverted grayscale (**a, b**) and 21-step color (**c, d**) FDG-PET images from a patient (ID#6) showing a metabolic response to lower-dose sunitinib in a right upper lobe lung lesion (circles). **a, c**: Baseline images prior to initiation of therapy with sunitinib. **b, d**: Follow-up images obtained after 28 days of treatment with lower-dose sunitinib. Circles mark the location of the tumor to distinguish it from the normal cardiac and hepatic uptake

**Fig. 2:**
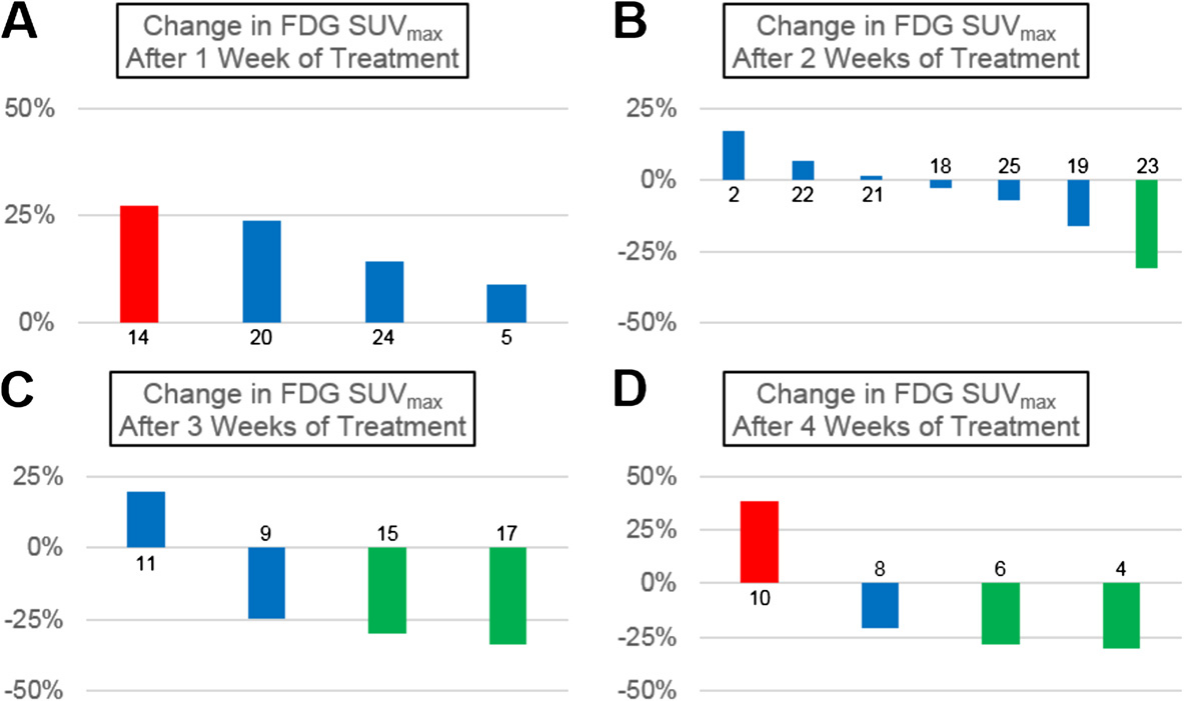
Metabolic tumor response to 37.5 mg daily sunitinib over time as measured with FDG-PET. Graphs illustrating the percent change in FDG SUV_max_ from pre-treatment baseline to after 1 (**a**), 2 (**b**), 3 (**c**), or 4 (**d**) weeks of sunitinib therapy in individual patients. There was a delayed metabolic response to continuous lower-dose sunitinib beginning after two weeks of treatment that was maintained throughout the duration of this study. Green bars represent metabolic response (≥25 % decrease in FDG SUV_max_), red bars represent metabolic progression (≥25 % increase in FDG SUV_max_), and blue bars represent stable disease (<25 % change in SUV_max_). The bars are labeled with the corresponding patient study identification number

The target lesions identified on previous clinical imaging were associated with FDG uptake on both baseline and follow-up PET imaging in all patients (Fig. 1). The average SUV_max_ as baseline and follow-up for each subject is summarized in Table 2. A metabolic response (reduction in tumor metabolism of ≥ 25 % as measured with FDG-PET) was observed in 5/19 (26 %) of patients; with 0, 1, 2, and 2 patients at 1, 2, 3, and 4 weeks, respectively (Fig. 2, Table 3). Metabolic disease progression (increase in tumor metabolism of ≥ 25 % as measured with FDG-PET) was observed in 2/19 (11 %) of patients; with 1, 0, 0, and 1 patients at 1, 2, 3, and 4 weeks, respectively. Interestingly, tumor metabolic response appeared to be delayed and was not observed until after at least 2 weeks of continuous lower-dose sunitinib therapy. This effect was confirmed as a significant trend in metabolic response with increasing weeks of treatment (cohorts) (likelihood ratio chi-square = 4.00, *p* = 0.045).

**Table 2:** FDG-PET and FLT-PET Results

		FDG-PET			FLT-PET		
Subject #	Treatment Cohort	Baseline Average SUV_max_	Follow-up Average SUV_max_	Metabolic Response	Baseline Average SUV_max_	Follow-up Average SUV_max_	Proliferative Response
14	Week 1	4.9	6.2	PD	6.9	8.4	SD
24	Week 1	5.1	5.8	SD	5.5	5.9	SD
20	Week 1	5.2	6.4	SD	7.4	5.3	PR
5	Week 1	4.9	5.3	SD	4.5	2.8	PR
2	Week 2	4.7	5.6	SD	4.7	4.5	SD
21	Week 2	3.4	3.5	SD	6.2	3.1	PR
22	Week 2	8.7	9.3	SD	6.8	6.7	SD
19	Week 2	17.9	15.0	SD	7.8	5.6	PR
23	Week 2	18.1	12.5	PR	5.0	5.3	SD
25	Week 2	9.6	9.0	SD	5.8	6.2	SD
18	Week 2	12.6	12.2	SD	8.4	5.1	PR
9	Week 3	21.0	15.8	SD	N/A	5.3	N/A
15	Week 3	6.0	4.2	PR	3.4	2.5	PR
16	Week 3	10.9	N/A	N/A	9.7	12.7	PD
11	Week 3	2.7	3.2	SD	1.3	1.4	SD
17	Week 3	1.5	1.0	PR	0.7	0.4	PR
6	Week 4	7.3	5.2	PR	5.1	2.1	PR
8	Week 4	8.9	7.1	SD	14.6	7.5	PR
10	Week 4	4.9	6.7	PD	5.1	5.9	SD
4	Week 4	7.0	4.9	PR	5.3	5.0	SD

**Table 3:** PET Imaging Response Rates

Treatment	Metabolic Assessment			Proliferative Assessment		
Cohort	PR	SD	PD	PR	SD	PD
Week 1	0/4 (0 %)	3/4 (75 %)	1/4 (25 %)	2/4 (50 %)	2/4 (50 %)	0/4 (0 %)
Week 2	1/7 (14 %)	6/7 (86 %)	0/7 (0 %)	3/7 (43 %)	4/7 (57 %)	0/7 (0 %)
Week 3	2/4 (50 %)	2/4 (50 %)	0/4 (0 %)	2/4 (50 %)	1/4 (25 %)	1/4 (25 %)
Week 4	2/4 (50 %)	1/4 (25 %)	1/4 (25 %)	2/4 (50 %)	2/4 (50 %)	0/4 (0 %)

### Early assessment of proliferative response with semi-quantitative FLT-PET

**Fig. 3:**
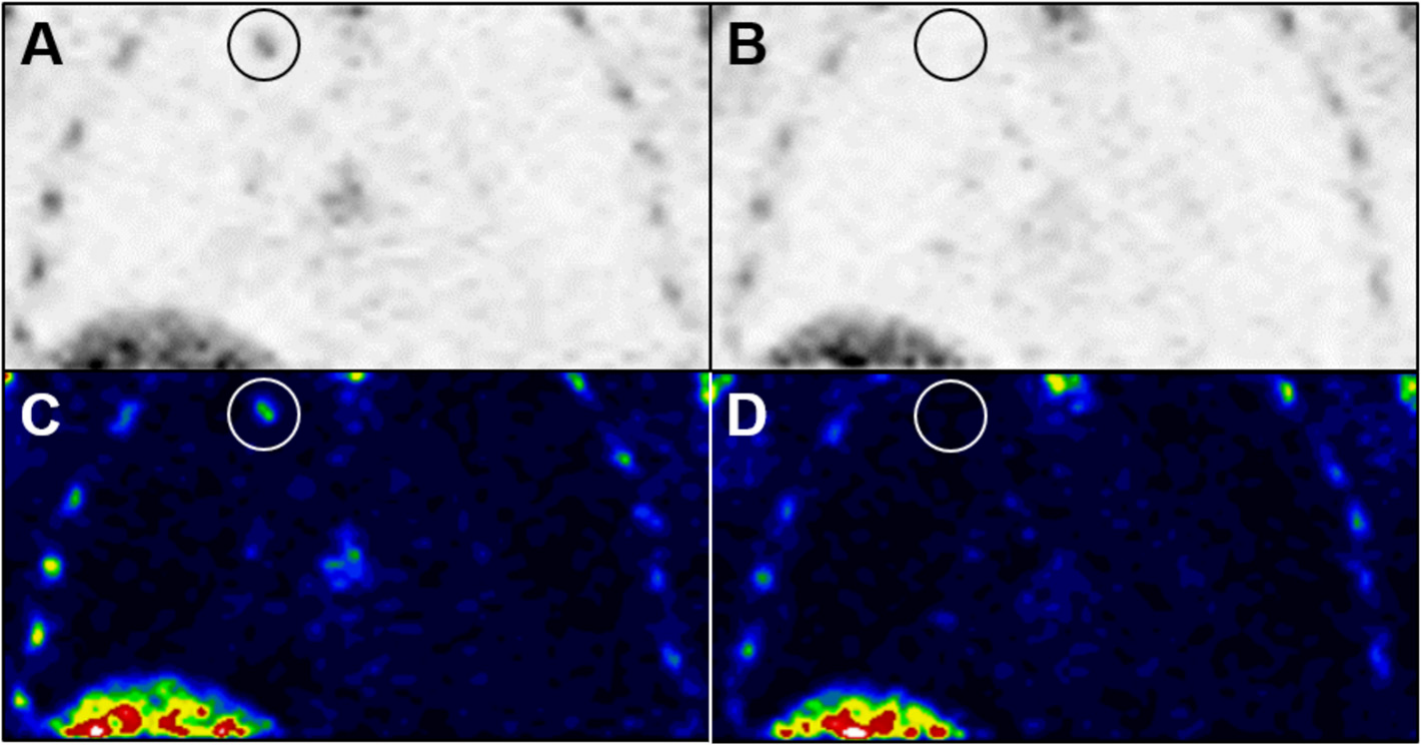
FLT-PET images illustrating a proliferative response to 37.5 mg daily sunitinib treatment. Single slice coronal inverted grayscale (**a, b**) and 21-step color (**c, d**) FLT-PET images from the same patient as in Fig. 1 (ID#6) showing a proliferative response to lower-dose sunitinib in a right upper lobe lung lesion (circles). **a**, **c**: Baseline images prior to initiation of therapy with sunitinib. **b**, **d**: Follow-up images obtained after 28 days of treatment with lower-dose sunitinib. Circles mark the location of the tumor to distinguish it from the normal skeletal and hepatic uptake

**Fig. 4:**
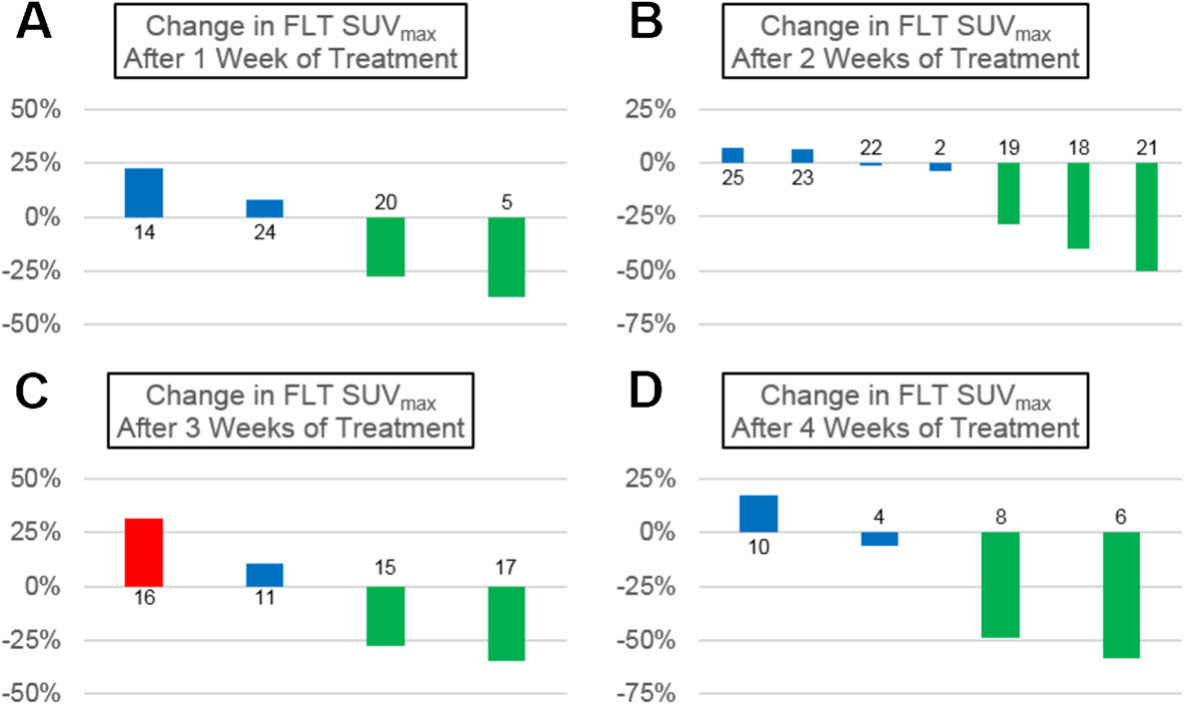
Proliferative tumor response to 37.5 mg daily sunitinib over time measured with FLT-PET. Graphs illustrating the percent change in FLT SUV_max_ from pre-treatment baseline to after 1 (**a**), 2 (**b**), 3 (**c**), or 4 (**d**) weeks of sunitinib therapy in individual patients. There was an immediate proliferative response to continuous lower-dose sunitinib that was maintained throughout the duration of this study. Green bars represent proliferative response (≥25 % decrease in FLTSUVmax), red bars represent proliferative progression (≥25 % increase in FLT SUV_max_), and blue bars represent stable disease (<25 % change in SUV_max_). The bars are labeled with the corresponding patient study identification number

Similarly to FDG, the target lesions identified on previous clinical imaging were associated with FLT uptake on both baseline and follow-up PET imaging in all patients (Fig. 3). A partial proliferative response (reduction in tumor proliferation of ≥ 25 % as measured with FLT-PET) was observed in 9/19 (47 %) of patients; with 2, 3, 2, and 2 patients at 1, 2, 3, and 4 weeks, respectively (Fig. 4, Table 3). Proliferative disease progression (increase in tumor proliferation of ≥ 25 % as measured with FLT-PET) was observed in 1/19 (5 %) of patients; with 0, 0, 1, and 0 patients at 1, 2, 3, and 4 weeks, respectively. Unlike metabolic responses measured with FDG-PET, tumor proliferative response to continuous lower-dose sunitinib was not delayed as it was observed at the earliest interval of follow-up (1 week) and appeared to remain stable throughout the entire period of observation for this study with similar response rates for each cohort. In contrast to FDG-PET, there is no trend in proliferative response with FLT-PET across cohorts with increasing weeks of treatment (likelihood ratio chi-square = 0.01, *p* = 0.93).

### Baseline FDG and FLT average SUV_max_ as predictors of survival

**Fig. 5:**
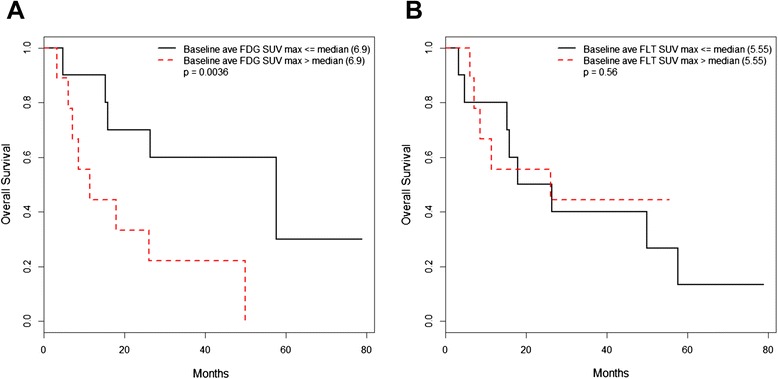
Baseline average FDG-PET SUV_max_ is predictive of overall survival. Kaplan-Meier plots of baseline FDG average SUV_max_ (**a**) and baseline FLT average SUV_max_ (**b**) as a predictor of overall survival. The baseline FDG average SUV_max_ was predictive of overall survival with higher SUV_max_ values correlated with shorter overall survival (*p* = 0.0036). Conversely, the baseline FLT average SUV_max_ did correlate with overall survival (*p* = 0.56)

As expected, higher baseline FDG-PET average SUV_max_ values correlated with shorter overall survival (*p* = 0.0036, Table 4, Fig. 5a,), indicating prognostic value for early assessment with FDG-PET in this study of patients with metastatic RCC. In contrast, baseline FLT-PET did not have prognostic value in this study as the baseline FLT-PET average SUV_max_ values did not correlate with overall survival (*p* = 0.56, Table 4, Fig. 5b).

**Table 4:** Results of Cox Proportional Hazards Models – Continuous Predictors

Predictor	Coefficient	Hazard Ratio	Robust Standard Error (se)	Z score	P value (Robust Wald Statistic)	Multiple comparison adjusted p-value (Holm procedure)
Baseline average FDG SUV_max_	0.100479	1.105701	0.032182	3.122199	0.001795055	0.0036
Baseline average FLT SUV_max_	0.033022	1.033573	0.056237	0.587196	0.557072332	0.56

## Disscussion

Here we provide evidence that PET imaging may be a valuable tool in the early assessment of response to continuous treatment with sunitinib in metastatic RCC. Despite the small sample size in this exploratory study, baseline FDG-PET SUV_max_ was prognostic of overall survival. Conversely, correlations of baseline FLT-PET semi-quantitative measures with overall survival were not statistically significant in this study. This would suggest that tumor metabolism may be more useful for the assessment of disease burden and/or tumor aggressiveness than the rate of proliferation in this setting. In other studies however, semi-quantitative PET measures such as SUV_max_ have shown prognostic value in many cancer types using multiple radiotracers [38]. Additional PET imaging studies in metastatic RCC comparing FDG-PET and FLT-PET will be needed before definitive conclusions can be drawn.

Reductions in both tumor metabolism and proliferation in response to continuous lower-dose sunitinib were observed with PET using FDG and FLT, respectively. Interestingly, the timing of these responses were somewhat offset. Proliferative response to continuous lower-dose sunitinib was noted early in the treatment course, after 1 week of therapy (the earliest interval of observation), and remained constant throughout the duration of the study. Conversely, metabolic responses were relatively delayed, not being observed until after at least 2 weeks of treatment. This suggests that inhibition of VEGF and PDGF signaling with sunitinib exerts an early effect on tumor proliferation, [39] which is then followed by a reduction in tumor metabolism. However, due to the fact that patients did not undergo imaging at multiple time points during treatment in this study and the small number of subjects, we cannot rule out that the chronology of the PET findings were an artifact of individual patient responses. Farnebo, et al. did observe a maintained reduction in FDG uptake in patients imaged after 2 and 4 weeks of treatment, indicating that the lack of repeat imaging in our study could be biasing our results, however, that study included patients treated with other tyrosine kinase inhibitors and their data were not stratified based upon treatment agent [24]. Of note, our study assessed only the earliest response to treatment (1–4 weeks) and it is not known whether or not the reduction in tumor metabolism is transient or maintained, or if later effects on tumor proliferation also occur. Previous work evaluating response to treatment with sunitinib in metastatic RCC has shown that reductions in tumor metabolism detected with FDG-PET are maintained for up to 16 weeks [20–22]. However, the prevalence of metabolic responders is reduced at later time points,[20] indicating that tumor resistance to sunitinib may develop after the period of observation captured in our study and that early response may not be indicative of overall outcome. Liu, et al. conducted a study in which patients were imaged with FLT-PET after either 2 or 4 weeks of treatment with sunitinib, however, this study focused on the effects of the standard drug holiday on tumor proliferation and revealed a significant increase in tumor proliferation off drug [30].

The post-treatment imaging and response assessment could not be compared to survival due to the limited patient numbers and lack of compliance in continuing treatment after the follow-up imaging time point. Many patients discontinued sunitinib therapy shortly after their follow-up PET imaging and went on other subsequent therapies. For these reasons, it cannot be assessed whether the differences seen in early imaging response with FDG-PET and FLT-PET are predictive of survival. It is possible that FLT-PET and FDG-PET can still be used as pharmacodynamic biomarkers at the appropriate timing during treatment without predicting the overall survival.

An obvious limitation of our study was the use of a PET-only tomograph. We were not able to assess whether or not response to treatment as measured with PET corresponded to RECIST changes with anatomic imaging as CT scans were not performed at the time of PET imaging. Standard of care clinical CTs were obtained at varying intervals, however, these images were obtained weeks to months after the conclusion of our PET imaging study as some patients did not continue treatment with sunitinib and those CTs were obtained prior to beginning a new type of therapy.

## Conclusions

The results of this study, while exploratory in nature, suggest that PET imaging with both FDG and FLT has utility in the early evaluation of response to treatment in metastatic RCC. While FLT-PET could be used to identify response as early as 1 week after starting treatment, our results suggest FDG-PET is more effective at later time points of 3–4 weeks. Additional studies with longer treatment duration are needed to more fully characterize the metabolic and proliferative responses in the context of predicting post treatment survival. Despite the limited subject numbers of this study, the baseline FDG-PET average SUV_max_ was a prognostic predictor of survival. More broadly, this study underscores the importance for the inclusion of early PET imaging assessments in clinical studies to define the best imaging approach for early therapeutic assessment in targeted therapies. The on-going evolution of PET/CT imaging will continue to improve the assessment of physiologic and biologic parameters as well as to facilitate improved accuracy of lesion size measurement with concurrent CT imaging. Defining the best imaging modalities for early therapeutic assessment of response or treatment failure will allow for a more streamlined, cost effective and personalized approach to cancer patient care.

## Abbreviations

CT: (X-ray computed tomography)
FDG: (18 F-fluorodeoxyglucose)
FLT: (18 F-fluorothymidine)
MRI: (Magnetic resonance imaging)
PDGF: (Platelet-derived growth factor)
PET: (Positron emission tomography)
RCC: (Renal cell carcinoma)
SUV: (Standardized uptake volume)
SUV: (Maximum standardized uptake volume)
VEGF: (Vascular endothelial growth factor)
